# The Trip Adviser guide to the protein science world: a proposal to improve the awareness concerning the quality of recombinant proteins

**DOI:** 10.1186/1756-0500-7-585

**Published:** 2014-09-01

**Authors:** Mario Lebendiker, Tsafi Danieli, Ario de Marco

**Affiliations:** Protein Expression and Purification Facilities, Wolfson Centre for Applied Structural Biology, Givat-Ram Campus - The Hebrew University of Jerusalem, 91904 Jerusalem, Israel; Department of Biomedical Sciences and Engineering, University of Nova Gorica, Glavni Trg 9, SI-5261 Vipava, Slovenia

**Keywords:** Data annotation, Data reproducibility, Protein quality

## Abstract

In many research articles, where protein purification is required for various assays, (protein-protein interactions, activity assays, etc.), we always have access to the final results, but seldom have access to the raw data required for an accurate evaluation of the protein quality. This data is extremely important on one hand to critically evaluate the quality of the proteins used in the described research and, on the other hand, to allow other laboratories to safely use the described procedure in a reproducible manner. We herby propose to include a standardized methodology that can easily be incorporated in research papers. Moreover, this methodology can be utilized as a “quality control” ladder, where the more information given, will lead to a higher ranking of the article. This “quality control” stamp will allow researchers retrieving relevant and useful materials and methods in the field of protein research.

**Electronic supplementary material:**

The online version of this article (doi:10.1186/1756-0500-7-585) contains supplementary material, which is available to authorized users.

## Commentary

At least 50% of the published studies –even in the most prestigious journals- cannot be reproduced [1–8]. Designing, writing, reviewing, publishing, and referring to data obtained under misleading experimental conditions is clearly an expensive and unproductive procedure for all the actors involved in the scientific system. Moreover, the accumulated errors are amplified by each secondary publication that was based on non-reproducible data.

Minimal Information (MI) checklists have been proposed for standardization of experimental description but a general drawback of these platforms is that they have been primarily conceived for simplifying the bioinformatics (re)use of experimental data. This effort is meaningful because metadata analysis of standardized datasets represents a valuable source of information and maximizes the usage of already existing results [9–11]. Nevertheless, annotation following mandatory guidelines is often cumbersome and conflicting MI checklists have been proposed, despite the simplification efforts made by the community [12–14]. Moreover, only a few cases of MI checklists focus on methodologies for recombinant protein production and quality evaluation [15].

Protein production at lab scale is a straight-forward procedure. Nevertheless, each step implies making choices, providing controls, and dealing with the evident as well as the unappreciated pitfalls of the technology, such as changes in protein expression, physical and chemical alterations in protein structure, aggregation, and proteolysis. Since protein production is very often not the aim of most research projects, but simply the way to obtain intermediate reagents to start a research project, poor protein quality will undermine the robustness of complex multidisciplinary efforts. At the same time, general (cell) biologists are less aware of protein quality than specialists such as crystallographers, enzymologists, or protein chemists and biotechnologists. Therefore, we wish to propose a methodology for improving the qualitative evaluation of their proteins to researchers who are not “protein production specialists”.

Based on many years of experience in the protein production field, we would like to propose a practice that should simplify the assessment of the experimental set based on a flowchart for initial evaluation of experimental steps in protein production together with the corresponding data to append as Additional file 1 according to the guidelines of established initiatives such as Biosharing/MIBBI Foundry.

The protein production flowchart (Additional file 1) should help following the design of the protein production protocol outlining the critical points and to standardize and reproduce the results in other laboratories [16, 17]. We suggest editors and reviewers to encourage (not compel) researches to fill as many as possible of the listed requests (following the already available standards) to acquire the necessary information for the reliable evaluation of the proposed work. Clearly, the set of relevant data will change according to the final use of the protein and, therefore, there is no reason for mandatory universal guidelines (Table 1).

**Table 1:** **The most basic requirements for evaluating protein quality**

In-deep protein biophysical characterization needs specific expertise and specialized equipment, but any biology lab should be able to assess the produced proteins using to at least two complementary techniques:	
1.	PAGE-SDS provides multiple information regarding the quality of the protein such as the presence of degradation products as well as the absence of protein contamination.
2.	Analytical size exclusion chromatography (SEC) [18] provides information regarding the correct oligomeric structure of the protein and the absence of soluble aggregates that can cause non-specific results in downstream experiments.

Some editors might even consider attaching a special section of comments to the electronic version of the paper, allowing peers to grade the quality of the described protein production procedure, similar to sites such as TripAdvisor and others.

## Electronic supplementary material

Additional file 1: **Flowchart corresponding to a basic lab-scale protein production protocol.** Process evaluation check-list allows for the precise identification of the steps and illustrates for each module the meaningful actions necessary to characterize the proteins used as reagents in biological experiments. Click the links on the image to obtain specifications and instructions. When available, module annotations should be completed according to the guidelines of accepted MI platforms: Biosharing/MIBBI Foundry http://www.biosharing.org/standards/mibbi. For instance: protein _purification_chromatography; http://wwwdev.ebi.ac.uk/micheckout/checkout/html?output-type=view_as_html_table&accessions=Gel_electrophoresis. (PDF 418 KB)
