# Supplementary material for: The Trip Adviser guide to the protein science world: a proposal to improve the awareness concerning the quality of recombinant proteins
Source: BMC Res Notes. 2014 Sep 1;7:585. doi: 10.1186/1756-0500-7-585 (PMC4161829; doi:10.1186/1756-0500-7-585)
Supplement: Supplementary file 1 — Additional file 1: Flowchart corresponding to a basic lab-scale protein production protocol. Process evaluation check-list allows for the precise identification of the steps and illustrates for each module the meaningful actions necessary to characterize the proteins used as reagents in biological experiments. Click the links on the image to obtain specifications and instructions. When available, module annotations should be completed according to the guidelines of accepted MI platforms: Biosharing/MIBBI Foundry http://www.biosharing.org/standards/mibbi. For instance: protein _purification_chromatography; http://wwwdev.ebi.ac.uk/micheckout/checkout/html?output-type=view_as_html_table&accessions=Gel_electrophoresis. (PDF 418 KB) [file 13104_2014_3115_MOESM1_ESM.pdf]

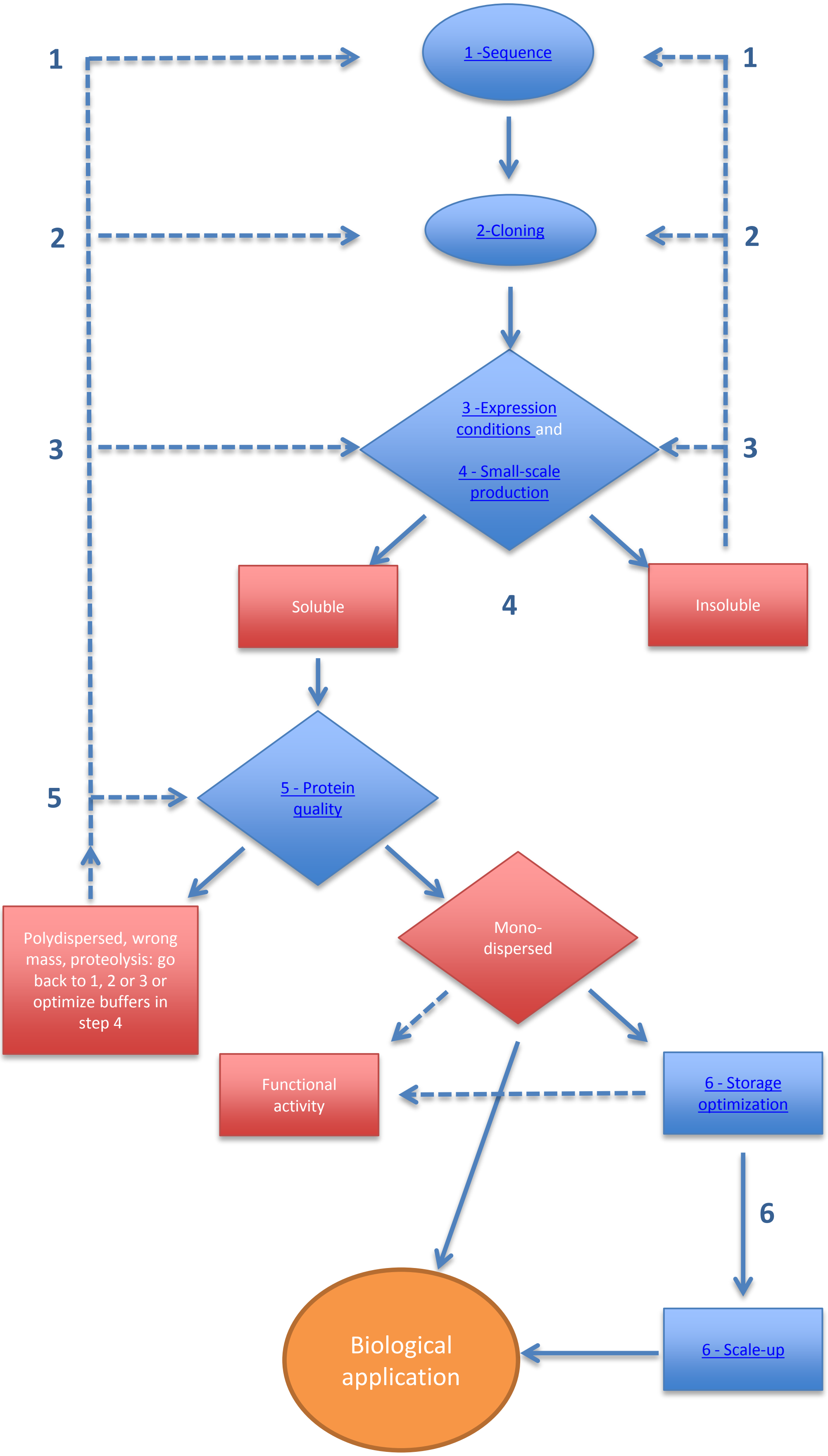

1
1 -Sequence
1
2
2-Cloning
2
3
3 -Expression conditions and
4 - Small-scale production
3
Soluble
4
Insoluble
5
5 - Protein quality
Polydispersed, wrong mass, proteolysis: go back to 1, 2 or 3 or optimize buffers in step 4
Mono-dispersed
Functional activity
6 - Storage optimization
6
Biological application
6 - Scale-up

Sequence:

a) Gene ID from a known database (PubMed tools, etc.)
b) Domain determination of the specific gene ID, (i.e. aa 48-330 from Id xxx)
c) Specific elements (Signal peptide, trans-membrane sequence, cellular localization, specific protease sites, etc.)
d) Fusion tags
e) Protease sites for tag removal
f) Full translated region, DNA and AA sequences

Cloning

a) Vector name either commercial or from known database (such as link to AddGene - vector sequence, annotated map with resistance, ori, cloning methodology, etc.)

b) Cloning method (ligation, recombination, etc.)

c) DNA region into which the sequence described is inserted

d) Bacterial strain used for storage and propagation (commercial producer, genotype, cat. Number, or database)

Expression conditions

a) Expression system (Host specification such as: bacterial strain, cell line, cat. Number, link to database)
b) Growth and induction conditions: temperature, media and supplements, rpm, oxygenation, inducer, time, quantity)
c) Instrumentation used (Bioreactors, shakers, flasks, etc.)

Small scale production: Soluble / Insoluble

a) Cell lysis: buffers and lysis procedure
b) Purification conditions: resin, buffers, yield
c) PAGE-SDS analysis: method (sup vs. pellet, and/ or affinity binding: analyzed on Coomasie and/or Western blot)

Protein quality

Basic requirements for evaluating monodispersity and degradation

a) PAGE-SDS (coomassie and/or Western blot)
b) Analytical size exclusion chromatography (SEC)

Additional information for evaluating protein quality

c) Functional activity: short description of the assay and results

d) Others: Circular Dicroism (CD), Optical Density (OD) spectrum, Mass Spectrum (MS), analytical ion exchange (IEX) , Reverse phase chromatography (RPC), Dynamic and Static Light Scattering (DLS and SLS), Size Exclusion Chromatography - Multi Angle (Laser) Light Scattering Method (SEC-MALS), etc

Scale-up and storage conditions

a) Initial growth volume (OD, cell mass etc.)
b) Lysis conditions: buffers, additives, lysis methodology, clarification procedure
c) Chromatography: Resin supplier, column volume, buffers, loading, washing, and elution conditions. Final yield
d) Description of successive chromatographic steps as before
e) Storage conditions: protein concentration, storage buffer, temperature
f) Quality control: PAGE-SDS analysis , analytical SEC , protein quantification method, etc
